# Machine Learning and Blood-Targeted Proteomics Enable Early Prediction and Etiological Discrimination of Hypertensive Pregnancy Disorders

**DOI:** 10.3390/ijms27031402

**Published:** 2026-01-30

**Authors:** Natalia Starodubtseva, Alisa Tokareva, Alexey Kononikhin, Anna Bugrova, Maria Indeykina, Evgenii Kukaev, Alina Poluektova, Alexander Brzhozovskiy, Evgeny Nikolaev, Gennady Sukhikh

**Affiliations:** 1 V.I. Kulakov National Medical Research Center for Obstetrics Gynecology and Perinatology, Ministry of Healthcare of Russian Federation, 117997 Moscow, Russia; a_tokareva@oparina4.ru (A.T.);; 2 Project Center of Omics Technologies and Advanced Mass Spectrometry, 121205 Moscow, Russia; 3 Emanuel Institute of Biochemical Physics, Russian Academy of Sciences, 119334 Moscow, Russia; 4 V.L. Talrose Institute for Energy Problems of Chemical Physics, N.N. Semenov Federal Research Center for Chemical Physics, Russian Academy of Sciences, 119334 Moscow, Russia; 5 Moscow Center for Advanced Studies, 123592 Moscow, Russia; 6 Department of Obstetrics, Gynecology, Perinatology and Reproductology, Institute of Professional Education, Federal State Autonomous Educational Institution of Higher Education I.M. Sechenov First Moscow State Medical University of the Ministry of Health of the Russian Federation, 119991 Moscow, Russia

**Keywords:** preeclampsia, gestational arterial hypertension, chronical arterial hypertension, proteome, serum, pregnancy, machine learning

## Abstract

Imperfect first-trimester screening for hypertensive disorders of pregnancy (HDP) means many high-risk women miss the window for preventive aspirin, and the biological heterogeneity of HDPs is overlooked. This study aimed to leverage first-trimester serum proteomics to create a more precise tool for predicting preeclampsia (PE) and differentiating it from other HDPs. A prospective nested case–control study (*n* = 172) was conducted using targeted liquid chromatography-multiple reaction monitoring-mass spectrometry (LC-MRM-MS) proteomic profiling of 115 proteins. Machine learning (ML) methods were used to develop classifiers from the proteomic data. The signature predictive of PE was characterized by dysregulation of the complement and coagulation cascades (*F10*, *C8A*, *C1QA*, *SERPING1*, *VTN*). The profile differentiating gestational hypertension (GAH) from chronic hypertension (CAH) was linked to lipid metabolism (*HRG*, *APOA4*, *APOC2*). An 18-protein support vector machine (SVM) model for predicting PE demonstrated exceptional performance, with 94% sensitivity and 100% specificity, significantly outperforming the standard Fetal Medicine Foundation (FMF) screening algorithm. Pathway analysis confirmed that PE is associated with early activation of innate immunity and coagulation pathways, while GAH is linked to a pregnancy-induced metabolic response. A targeted serum proteomic combined with ML approach represents a new perspective diagnostic tool with strong potential to personalize monitoring for women at the highest risk for specific hypertensive pregnancy complications.

## 1. Introduction

Hypertensive disorders of pregnancy (HDP)—including chronic hypertension (CAH), gestational hypertension (GAH), and preeclampsia (PE)—are leading causes of maternal and perinatal morbidity and mortality worldwide. PE, the most severe form, is characterized by systemic endothelial dysfunction and placental malperfusion, often leading to multi-organ complications [1,2]. While low-dose aspirin can reduce the incidence of preterm PE by over 60%, its efficacy hinges on early and accurate identification of high-risk women [3].

The first-trimester screening algorithm developed by the Fetal Medicine Foundation (FMF), which combines maternal factors, mean arterial pressure (MAP), uterine artery Doppler, and serum biomarkers (placental growth factor, PIGF and pregnancy-associated plasma protein-A, PAPP-A), demonstrates strong predictive performance in research settings. However, its sensitivity in real-world cohorts is more modest, ranging from 63% to 71% [4,5,6]. This performance gap, potentially attributable to operator-dependent variability in Doppler measurements [7], means that 25–40% of women who develop preterm PE are missed and do not receive aspirin prophylaxis.

The clinical challenge is compounded by the fact that HDPs are a spectrum of disorders with different underlying causes and risks [8,9,10]. While GAH increases the risk of severe maternal morbidity by 50%, CAH without PE presents a risk profile similar to normotensive pregnancies [11]. Furthermore, CAH is independently linked to placental complications [12]. The proven benefit of early blood pressure control, as demonstrated in the CHAP trial, highlights the critical need to identify and differentiate these conditions early [13]. Current screening falls short in this regard, as it is primarily designed to predict placental PE and cannot distinguish between the varied etiologies across the HDP spectrum.

High-throughput proteomics offers a promising path forward, with several studies demonstrating that first-trimester alterations in the maternal serum proteome can predict PE long before clinical onset [14,15,16,17]. However, most proteomic investigations to date have treated HDP as a unified group or compared PE only to normotensive controls, without differentiating between CAH and GAH [18,19].

To address this gap, this study aimed to derive and validate a first-trimester maternal serum proteomic signature capable of not only predicting HDP but also differentiating their underlying etiologies. By applying a rigorously validated targeted liquid chromatography-multiple reaction monitoring mass spectrometry (LC-MRM-MS) platform [20] together with machine learning, this approach successfully distinguished PE from GAH, pre-existing CAH, isolated intrauterine growth restriction (IUGR), and normotensive pregnancy.

## 2. Results

### 2.1. Analysis of First-Trimester Predictors and Delivery Outcomes

**Figure 1:**
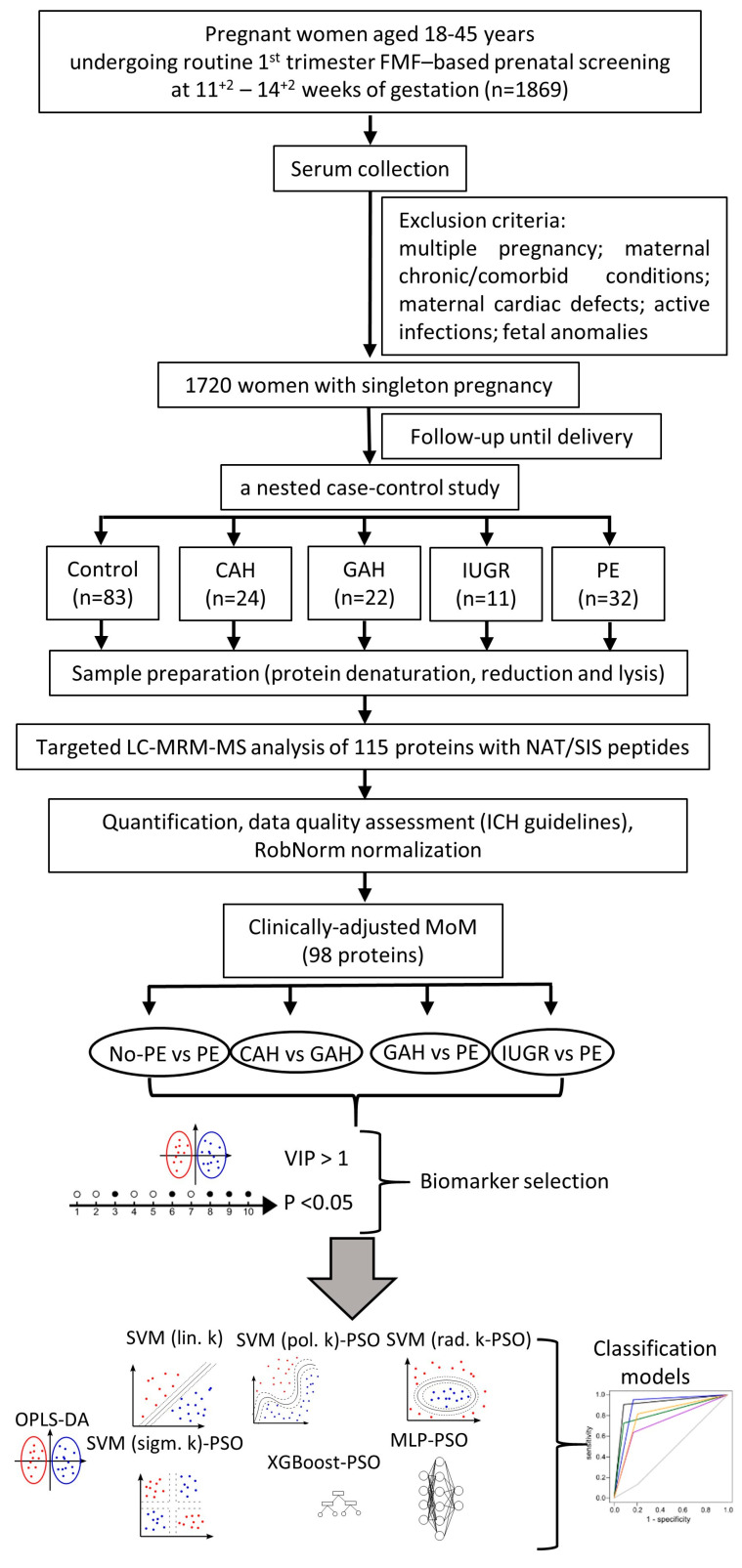
Overview of the experimental workflow from clinical sample collection to machine learning classifier development.

This prospective study included 172 women undergoing first-trimester prenatal screening at 11^+2^ to 14^+2^ weeks of gestation, comprising five groups: PE (*n* = 32), GAH (*n* = 22), CAH (*n* = 24), isolated IUGR (*n* = 11) without PE, and a control group (*n* = 83) (Figure 1). Within the PE group, most cases were late-onset (23/32, 72%) and moderate in severity (27/32, 84%); eleven women (34%) also had concomitant IUGR. Significant differences in maternal history, first-trimester biomarkers, and delivery outcomes were observed across these groups (Table S1).

Distinct maternal backgrounds were evident among the groups. A history of habitual miscarriage was significantly more common in the PE (38%, *p* < 0.001) and isolated IUGR (18%, *p* = 0.048) groups compared to the controls (0%). A prior history of PE was absent in both the control and GAH groups, but was present in the CAH (21%), isolated IUGR (18%), and PE (22%) groups (*p* < 0.001). Furthermore, a history of gestational diabetes mellitus (GDM) was significantly more frequent in women who developed PE (25%) compared to those without PE (1%) and to all other groups individually (*p* < 0.001). There were no differences between the groups in terms of parity (*p* > 0.05).

Women in the CAH and PE groups were significantly older than controls (mean 34.1 and 33.4 years vs. 30.3 years, *p* = 0.006 and *p* = 0.002, respectively). First-trimester body mass index (BMI) was also significantly higher in the CAH (median 24.9 kg/m^2^, *p* = 0.007) and GAH (median 23.5 kg/m^2^, *p* = 0.003) groups compared to controls (median 21.2 kg/m^2^).

Signs of early placental dysfunction were prominent in the PE group. These women exhibited significantly lower first-trimester PAPP-A (2.07 vs. 3.09 mIU/mL, *p* = 0.03) and PlGF (15.7 vs. 26.6 pg/mL, *p* < 0.001), alongside a higher UtA-PI (2.00 vs. 1.56, *p* < 0.001) compared to controls. The CAH and GAH groups also showed significantly lower adjusted PlGF levels (0.63 and 0.58 multiples of the median (MoM), respectively) than controls (0.76 MoM, *p* < 0.05). Consequently, a high risk for PE from first-trimester screening was significantly more common in all hypertensive groups (CAH 29%, GAH 23%, PE 53%) than in controls (1%, *p* < 0.001).

Notably, women with isolated IUGR and PE showed no significant differences in their medical history or most first-trimester screening parameters. The key exception was adjusted PlGF, which was significantly lower in the PE group (0.56 MoM) than in the isolated IUGR group (0.89 MoM, *p* = 0.005) (Table S2). In contrast, a direct comparison between the CAH and GAH groups revealed no statistically significant differences in their clinical first-trimester parameters (Table S3).

As expected, blood pressure was significantly elevated in all hypertensive disorders compared to controls, both in the first trimester and before delivery (*p* < 0.05). The PE and CAH groups presented the most severe hemodynamic profile, with the highest first-trimester MAP (91 and 93 mm Hg, respectively, *p* < 0.001) and the highest systolic/diastolic pressures before delivery (135/89 mm Hg and 123/80 mm Hg, *p* < 0.001 vs. controls). Moreover, an increase in blood creatinine, indicative of renal endothelial dysfunction, was found exclusively in the PE group (80.8 µM/L vs. 67.7 µM/L in controls, *p* < 0.001).

Evidence of placental insufficiency in PE was also reflected in umbilical cord Doppler parameters, showing an elevated pulsatility index (0.95 vs. 0.78 in controls, *p* < 0.001) and a reduced cerebro-placental ratio (1.5 vs. 1.84 in controls, *p* < 0.001) at delivery (Table S1). Consequently, birth weight and Apgar scores were significantly lower in the placenta-associated pathology groups (PE and isolated IUGR) compared to all others (*p* < 0.001).

Direct comparisons further clarified the distinctiveness of these conditions. When compared to GAH, PE was associated with worse placental function (lower first-trimester PlGF, higher cord artery PI, and lower cerebro-placental ratio at delivery, *p* < 0.01), significantly lower placental weight (*p* < 0.001), and poorer neonatal outcomes (*p* < 0.01) (Table S4). Placental dysfunction in the PE group was further evidenced at delivery by a significantly reduced placental weight (362 g vs. 455 g in non-PE groups, *p* < 0.001) and a high rate of preterm birth (44% vs. 1%, *p* < 0.001). Emergency cesarean section was also significantly more frequent in the PE group (59%) than in the non-PE groups combined (13%, *p* < 0.001) (Table S5). In contrast, the GAH and CAH groups had rates of term delivery comparable to controls.

Notably, only 47% of women in the PE group received prophylactic doses of acetylsalicylic acid, which may indicate that in more than half of these cases the risk of developing PE had been assessed as low. Low-molecular-weight heparins were prescribed most frequently in the PE group compared with all other study groups (59%, *p* < 0.001). It should also be noted that at the time of sample collection, the use of antihypertensive medications was reported only in the chronic hypertension group (33%, *p* < 0.001).

The comparison of the PE group to all non-PE participants consolidated its identity as a severe placental disorder, linked to a high-risk maternal background, a severe course of pregnancy from the first trimester, and markedly worse perinatal outcomes (Table S5).

### 2.2. Targeted Proteome Profiling and Analytical Performance

Proteomic analysis quantified 115 proteins that met the pre-defined quality control criteria (R^2^ > 0.99, precision and accuracy < 20% in over five of seven standard levels and in more than 66% of QC samples; Table S6). Twelve proteins were excluded from further analysis due to concentrations below the LLOQ or upper the HLOQ in over 50% of study samples. These were: Alpha-1-antitrypsin, Apolipoprotein A-I, Cholesteryl ester transfer protein, Clusterin, Complement C1q subcomponent subunit B, Fibrinogen alpha chain, Fibrinogen beta chain, Fibrinogen gamma chain, Kallistatin, Keratin type I cytoskeletal 9, Peroxiredoxin-2, and Serum paraoxonase/lactonase 3.

RobNorm normalization significantly reduced the median between-batch coefficient of variation from 6.6% (IQR: 5.0–9.6%) to 6.3% (IQR: 4.6–9.1%; *p* < 0.001, Mann–Whitney U test; Figure S1). An additional five proteins—Inter-alpha-trypsin inhibitor heavy chain H1, Inter-alpha-trypsin inhibitor heavy chain H2, L-selectin, Serum amyloid A-1 and A-2 proteins, and Zinc-alpha-2-glycoprotein—were excluded due to a high coefficient of variation (CV ≥ 20%).

Consequently, 98 proteins were included in the final analysis (Figure 1). The dynamic range spanned five orders of magnitude, from Protein deglycase DJ-1 (*PARK7*) at 2.5 (1.8; 3.6) nM to Serum Albumin (*ALB*) at 4.6 × 10^5^ (4.2 × 10^5^; 5.0 × 10^5^) nM (Table S7, Figure S2). Following normalization and adjustment to MoM values, Carbon Anhydrase 1 (*CA1*) exhibited the lowest median level (0.67 MoM, IQR: 0.36–1.19), while Biotinidase (*BTD*) exhibited the highest (1.06 MoM, IQR: 0.94–1.15). Hemopexin (*HPX*) was the most stable protein with a CV of 9%, whereas Apolipoprotein(a) (*LPA*) showed the widest variation with a CV of 284% (Table S8, Figure S3).

Serum concentrations of PAPP-A measured by routine ELISA and LC-MRM-MS were strongly correlated (Pearson’s R = 0.61, *p* < 0.001). This correlation remained virtually unchanged following MoM transformation (R = 0.60, *p* < 0.001) (Table 1).

### 2.3. Hypertension-Associated Serum Proteins

Proteomic profiling identified distinct protein signatures associated with the different clinical groups (*n* = 172). When comparing the isolated IUGR and PE groups, serum concentrations of Coagulation factor X (*F10*), Complement component C8 alpha chain (*C8A*), Complement C1q subcomponent subunit A (*C1QA*), Alpha-2-HS-glycoprotein (*AHSG*), Complement C1q subcomponent subunit C (*C1QC*), and Attractin (*ATRN*) were statistically significantly higher in the IUGR group, whereas Vitronectin (*VTN*), Ig gamma-1 chain C region (*IGHG1*), Ficolin-3 (*FCN3*), Transthyretin (*TTR*), and Plasma protease C1 inhibitor (*SERPING1*) were significantly higher in the PE group (Figure S4A, Table S9). An Orthogonal Projections to Latent Structures-Discriminant Analysis (OPLS-DA) model demonstrated good discrimination power for separating the IUGR and PE groups with R^2^Y = 0.99 and Q^2^Y = 0.46 (Figure S4B, Table 2). The most important proteins for this separation, with a VIP > 1, included Apolipoprotein(a) (*LPA*), *F10*, *C8A*, *C1QA*, *AHSG*, *SERPING1*, *C1QC*, *CA1*, *IGHG1*, Beta-Ala-His dipeptidase (*CNDP1*), *VTN*, *FCN3*, and *TTR* (Figure S4C). Consequently, *F10*, *C8A*, *C1QA*, *AHSG*, *C1QC*, *SERPING1*, *TTR*, *IGHG1*, *VTN*, and *FCN3* were selected as key discriminatory protein features. An OPLS-DA model based specifically on these potential markers demonstrated better predictive power (Q^2^Y = 0.67) (Table 2).

In the comparison between GAH and CAH, concentrations of *HRG*, *APCS*, *APOA4*, and *SERPINA6* were statistically significantly higher, while *APOC2* and *A1BG* were statistically significantly lower in the GAH group (Figure S5A, Table S10). The OPLS-DA model based on the proteome showed high discrimination power (R^2^Y = 0.89) (Figure S5B, Table 2). The most important proteins in this model according to VIP analysis are listed in Figure S5C. The proteins *HRG*, *APCS*, *APOA4*, and *SERPINA6*, along with *APOC2* and A1BG, were identified as discriminative for women with GAH versus CAH. An OPLS-DA model constructed using these features showed a substantial improvement in predictive power (Q^2^Y from 0.14 to 0.55) (Table 2).

For the separation of PE from GAH, concentrations of *TTR*, *SERPING1*, *HPX*, *VTN*, *C1RL*, and *APOA2* were statistically significantly higher in the PE group, while concentrations of *F10*, *C8A*, *AHSG*, *C1QA*, *C1QC*, *VCAM1*, and *KNG1* were statistically significantly lower (Figure S6A, Table S11). The OPLS-DA model based on the proteome had high discriminative and predictive power (R^2^Y = 0.97, Q^2^Y = 0.72) (Figure S6B, Table 2). The most important serum proteins are shown in Figure S6C. The proteins *TTR*, *SERPING1*, *HPX*, *VTN*, *C1RL*, *F10*, *C8A*, *AHSG*, *C1QA*, *C1QC*, *VCAM1*, and *KNG1* were defined as the discriminative proteins. The OPLS model based on these features maintained high discriminative power (R^2^Y = 0.82, Q^2^Y = 0.77) (Table 2).

**Figure 2:**
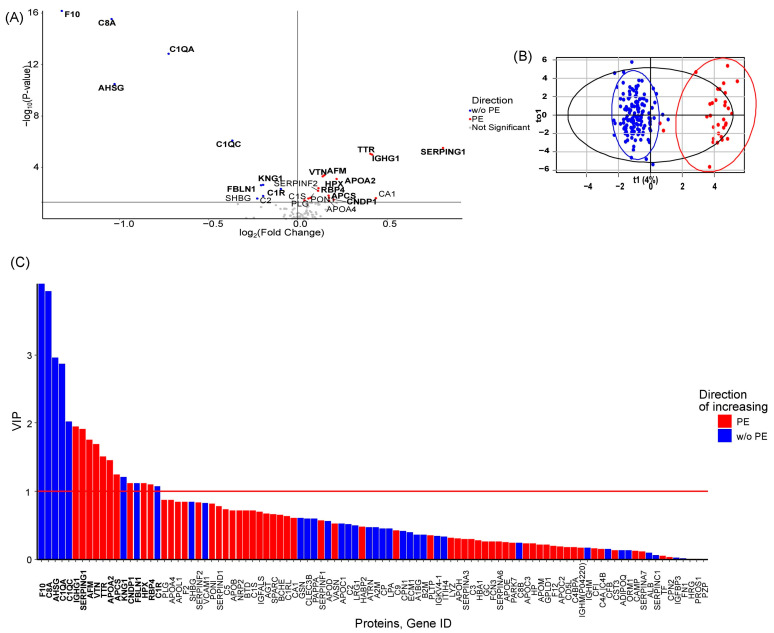
Discovery of first-trimester serum protein features predictive of PE. (**A**) Volcano plot illustrating differential protein abundance between the non-PE (*n* = 142) and PE (*n* = 32) groups. The fold change (PE/non-PE) is shown on the x-axis (log_2_ scale), and statistical significance (−log_10_ *p*-value, Mann–Whitney U test) is on the y-axis. Proteins identified as significant markers are labeled in bold. (**B**) OPLS-DA scores plot demonstrating the separation between the non-PE (blue) and PE (red) cohorts based on their proteomic profiles. (**C**) VIP scores for all proteins in the OPLS-DA model. Proteins with a VIP score > 1 (red line) significantly contribute to the separation and are highlighted in bold.

A consolidated analysis against non-PE groups defined a core first trimester serum protein signature for PE. Concentrations of *F10*, *C8A*, *AHSG*, *C1QA*, *C1QC*, *KNG1*, *FBLN1*, and *C1R* were statistically significantly decreased in PE, while concentrations of *SERPING1*, *IGHG1*, *TTR*, *AFM*, *VTN*, *APOA2*, *HPX*, *RBP4*, *APCS*, and *CNDP1* were statistically significantly increased (Figure 2A, Table S12). The OPLS-DA model based on the serum proteome showed high discriminative and predictive power (R^2^Y = 0.89, Q^2^Y = 0.79) (Figure 2B, Table 2). The most important proteins in the model according to VIP value are shown in Figure 2C. The final set of markers for the development of PE was defined as *F10*, *C8A*, *AHSG*, *C1QA*, *C1QC*, *FBLN1*, *KNG1*, *C1R*, *VTN*, *AFM*, *HPX*, *APOA2*, *RBP4*, *APCS*, *CNDP1*, *TTR*, *IGHG1*, and *SERPING1*. An OPLS-DA model based on these markers retained high discriminative and predictive power (R^2^Y = 0.84, Q^2^Y = 0.80) (Table 2).

**Figure 3:**
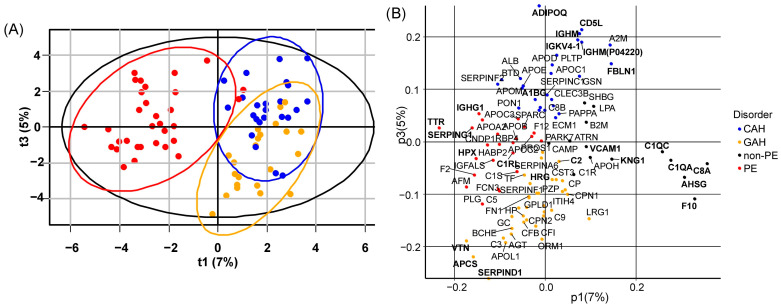
PLS-DA model distinguishing CAH (*n* = 24), GAH (*n* = 22), and PE (*n* = 32) samples. (**A**) Scores plot (components 1 vs. 3) showing sample distribution for CAH (blue), GAH (orange), and PE (red). (**B**) Corresponding loadings plot (components 1 vs. 3). Proteins with a VIP score > 1 are displayed in bold. Black labels indicate proteins that specifically differentiate PE from non-PE conditions (CAH and GAH) but do not distinguish between CAH and GAH.

**Figure 4:**
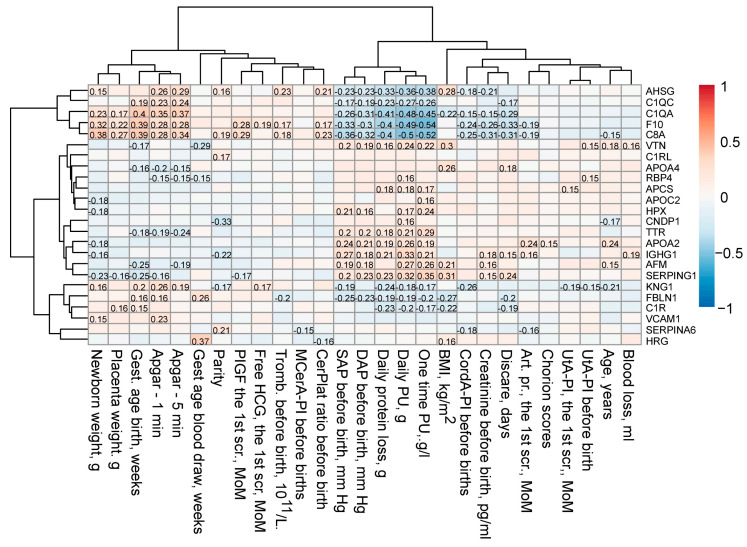
Association of protein markers with maternal, fetal, and neonatal clinical parameters. Cells display statistically significant correlation coefficients (*p* < 0.05).

A final PLS-DA model for discriminating CAH (*n* = 24), GAH (*n* = 24), and PE (*n* = 32) had high descriptive power (R^2^Y = 0.73) and medium predictive power (Q^2^Y = 0.38). GAH and CAH samples demonstrated good separation from PE samples but not from each other (Figure 3A). The loadings revealed that proteins significantly decreased (*p* < 0.05) in PE (*F10*, *AHSG*, *C8A*, *C1QA*, *C1QC*, *VCAM1*) were aligned with the “Non-PE” direction, while proteins significantly increased (*p* < 0.05) in PE (*TTR*, *SERPING1*, *IGHG1*, *HPX*, *C1RL*) were aligned opposite (Figure 3B, Table 3). Furthermore, proteins differentiating GAH from CAH (*p* < 0.05), such as *A1BG* (increased in CAH) and *HRG*/*APCS* (increased in GAH), were associated with the vector separating these two conditions (Figure 3, Table 3). Finally, these PE protein features showed significant associations with clinical parameters, correlating with adverse neonatal outcomes on one hand and maternal morbidity on the other (Figure 4).

### 2.4. Protein–Protein Interaction

The proteomic analysis of first-trimester maternal serum (*n* = 172) revealed significant insights into the molecular pathways associated with subsequent pregnancy complications. A predominant finding across most comparisons was the marked enrichment of pathways related to the complement system and coagulation cascades, underscoring their critical role in early disease pathophysiology. The discriminatory proteins for PE versus isolated IUGR, such as *VTN*, *FCN3*, *SERPING1*, *C8A*, *C1QC*, and *C1QA*, were predominantly localized to blood microparticles and the collagen-containing extracellular matrix (Figure S7A). Pathway analysis revealed a strong associated enrichment for the complement cascade, as detailed in Table S14. Furthermore, proteins including *SERPING1*, *C1QC*, and *C1QA* were linked to a pertussis pathway from the KEGG database, while a broader set comprising *VTN*, *TTR*, *FCN3*, *AHSG*, *SERPING1*, *C8A*, *C1QC*, and *C1QA* significantly enriched the Innate Immune System pathway from Reactome (Figure S7B, Table S13).

A distinct profile emerged for the discrimination between GAH and CAH. All six discriminatory proteins were extracellular, with five found in blood particles and the collagen-containing extracellular matrix (Figure S8A). The significantly enriched pathways were uniquely related to lipid metabolism, as proteins *APOA4* and *APOC2* were the sole drivers of pathways for familial hyperlipidemia and statin inhibition of cholesterol production in the Wikipathways database (Figure S8B, Table S14).

In the comparison of PE against GAH, the molecular signature again emphasized the involvement of blood microparticles and the extracellular matrix (Figure S9A). The pathway analysis confirmed a strong complement system activation, with six of the top ten enriched pathways being related to it (Table S15). This comparison was further distinguished by the significant enrichment of the kinin-kallikrein pathway by *SERPING1* and KNG1, and the intrinsic pathway of fibrin clot formation by *SERPING1*, *F10*, and *KNG1*, pointing to a specific dysregulation of inflammatory bradykinin signaling and coagulation. Associations with pertussis (*SERPING1*, *C1QC*, *C1QA*) and allograft rejection (*C8A*, *C1QC*, *C1QA*) pathways were also noted (Figure S9B, Table S15).

Finally, the broadest comparison of PE versus non-PE groups reinforced these findings, showing the most robust activation of complement and coagulation pathways. Eleven of the eighteen protein markers were contained in blood microparticles and eight in the collagen-containing extracellular matrix (Figure S10A). Seven of the ten most enriched pathways were associated with the complement system (Table S16). The analysis again confirmed the significant involvement of the intrinsic pathway of fibrin clot formation (*SERPING1*, *F10*, *KNG1*) and pathways for pertussis (*SERPING1*, *C1QC*, *C1QA*, *C1R*) and allograft rejection (*C8A*, *C1QC*, *C1QA*) (Figure S10B, Table S16). The recurrent enrichment of these specific pathways across the PE-specific comparisons suggests that dysregulated immune tolerance, inflammation, and coagulation are central features of preeclampsia’s early pathogenesis.

### 2.5. Classification Models

The list of serum proteins that demonstrated significant alterations (Table 3) in first trimester was used for building discrimination models based on OPLS-DA, Support Vector Machines (SVM, linear, polynomial, radial and sigmoid kernels), XGBoost and multilayer perceptron (MLP). SVM and OPLS-DA models demonstrated robust capability for discriminating between most clinical groups, with accuracies consistently above 90%. The exceptions were the comparisons between CAH and GAH, which were not discriminated as effectively (Table S17, Figure S11).

The sensitivity and specificity of the top-performing models for critical clinical distinctions are detailed in Table 4. Notably, SVM models employing polynomial and radial kernels perfectly distinguished IUGR from PE cases with 100% specificity and 94% sensitivity. The discrimination of PE from non-PE outcomes also achieved this high performance (100% specificity, 94% sensitivity) using similar SVM kernels. For distinguishing GAH from PE, an SVM model with a radial kernel showed balanced performance (95% sensitivity, 94% specificity). In contrast, the OPLS-DA model for differentiating CAH from GAH showed slightly lower, though still high, performance (91% sensitivity, 92% specificity).

The 18-protein SVM model demonstrated superior diagnostic accuracy compared to the standard FMF first-trimester prenatal screening for PE. The model achieved a sensitivity of 94% and a specificity of 100%, substantially outperforming the standard screen’s sensitivity of 53% and specificity of 91% (Figure S12, Table 5).

Notably, the standard screening yielded false positives in 1.2% of controls, 29.2% of chronic hypertension cases, and 22.7% of gestational hypertension cases, while failing to detect 47% of true PE cases. With an estimated PE prevalence of 4%, the FMF-based screening showed a positive predictive value (PPV) of 20% and a negative predictive value (NPV) of 98%. In contrast, the protein-based model achieved a PPV of 100% and an NPV of 99%, underscoring its enhanced reliability for both ruling in and ruling out the condition.

The first-trimester serum proteomic profile, comprising 115 proteins (*n* = 172), was successfully validated against an independent pilot cohort profiled with the same LC-MRM-MS platform (BAK-125 kit, MRM Proteomics, Montreal, QC, Canada), which measured 125 proteins (*n* = 40) [21]. The two datasets showed strong analytical concordance, with the 55 overlapping proteins exhibiting closely matched MoM-adjusted concentrations (Figure S13) and intermingled distribution in principal component space (Figure S14A).

This validation confirmed *CNDP1* and *TTR* as common, reproducible protein biomarkers for PE across both cohorts (Figure S14B,C). Furthermore, the diagnostic model developed in the larger cohort refined the predictive performance, increasing sensitivity to 94% (from 87%) and specificity to 100% (from 95%) compared to the earlier model [21].

## 3. Discussion

This prospective nested case–control study establishes that the first-trimester maternal serum proteome, analyzed via a rigorously validated targeted LC-MRM-MS approach, holds significant potential for the early prediction and etiological differentiation of major obstetric syndromes. We identified and validated distinct protein signatures capable of not only predicting the subsequent development of PE but also effectively distinguishing it from other disorders, including GAH, CAH and isolated IUGR. A key methodological strength was the derivation of clinically adjusted MoM values from a well-characterized reference group of 83 healthy pregnancies, which minimized physiological confounders and enhanced the clinical translatability of the findings [20].

The diagnostic performance of the models developed in this study underscores their clinical potential. The SVM models, particularly those with polynomial and radial kernels, achieved exceptional performance in distinguishing IUGR from PE and, most critically, to identify future PE cases within a general obstetric population. The 18-protein model achieved a sensitivity of 94% and a specificity of 100%, meaning it correctly identified nearly all PE cases while producing no false positives among controls or women with other hypertensive or growth-related conditions. This high specificity is crucial, as it could prevent unnecessary maternal anxiety and clinical interventions in low-risk women. The 94% sensitivity achieved here represents a substantial improvement over the current FMF screening algorithm, which failed to detect 47% of the PE cases in the study cohort.

This performance must be contextualized within the current landscape of PE prediction. A systematic review by Antwi et al. (2020) of 40 prediction models noted that most combine biomarkers with maternal characteristics, with PAPP-A and PlGF being the most common, and reported AUCs ranging from 0.70 to 0.98 [22]. The established FMF algorithm, which integrates maternal factors, mean arterial pressure, uterine artery Doppler, PAPP-A, and PlGF, achieves a detection rate of approximately 77% for preterm PE at a 10% false-positive rate in the ASPRE trial [23,24]. In contrast, the model developed here, with its superior sensitivity and specificity, outperforms this clinical benchmark.

Contemporary research also emphasizes the utility of accessible clinical data. For example, Chen et al. (2025) developed a pragmatic Hypertensive Disorders of Pregnancy (HDP) prediction model using first-trimester demographic and routine laboratory data (e.g., age, BMI, hemoglobin, lipids), achieving robust AUCs of 0.809 and 0.801 in modeling and validation cohorts, respectively [25]. Similarly, Zhou et al. (2024) developed a machine learning model using parameters like baseline BMI and blood pressure measured at 16–20 weeks, achieving a robust AUC of 0.85 [26]. While highly practical for resource-limited settings, Zhou et al.’s reliance on mid-pregnancy measurements shortens the window for early intervention. Although Chen et al.’s model uses first-trimester data, its foundation in standard clinical variables may not directly capture the underlying molecular pathophysiology of PE as effectively as a targeted proteomic approach [25].

This study’s lies in its ability not only to identify high-risk individuals but also to pinpoint the underlying cause of their risk. While conventional models group patients into a broad “high-risk” category, proteomic analysis reveals distinct biological pathways at work within this group. Notably, PE was marked by dysregulation in complement and coagulation cascades, in contrast to GAH and CAH, which were differentiated by alterations in lipid metabolism. These findings suggest that proteomic stratification can classify high-risk women by underlying etiology, enabling more personalized monitoring and management strategies long before clinical symptoms emerge [27].

Methodologically, this study addresses key critiques from Antwi et al., who highlighted frequent suboptimal calibration and validation in existing models [22]. Similarly to the approach of Chen et al., rigorous internal validation was conducted [25]. The validity of this work is further strengthened by the application of a targeted, quantitative LC-MRM-MS platform and the establishment of MoM values, offering a level of analytical rigor and pathophysiological insight that purely clinical models cannot provide.

The most prominent molecular theme identified in the data was early dysregulation of the complement system and coagulation cascades, a feature consistently observed across all PE-focused comparisons. The significant enrichment of pathways like “Complement cascade,” “Regulation of Complement cascade,” and “Classical antibody-mediated complement activation” as early as the first trimester suggests that aberrant immune activation at the maternal-fetal interface is a pivotal, initiating event in PE pathogenesis. This is corroborated by the enrichment of the “Innate Immune System” and “Allograft rejection” pathways, painting a picture of maladaptive immune response resembling failed fetal tolerance. Concurrently, the involvement of the “Intrinsic Pathway of Fibrin Clot Formation” and the “Kinin-Kallikrein system” links this immune dysregulation to a pro-coagulant and pro-inflammatory state, potentially driven by proteins like *SERPING1*, *F10*, and *KNG1*. This early co-activation provides a plausible molecular substrate for the subsequent endothelial dysfunction that defines clinical PE [28].

The analysis also successfully delineates disorder-specific proteomic profiles. For instance, comparison between PE and isolated IUGR revealed markedly distinct signatures: proteins like *F10*, *C8A*, *C1QA*, and *AHSG* were lower in PE, while *VTN*, *FCN3*, and *SERPING1* were elevated. This divergence points to separate pathogenic pathways from the first trimester, with PE exhibiting a more pronounced complement and inflammatory signature. Clinically, this translates to a more severe systemic response in PE, characterized by widespread endothelial activation and antiangiogenic imbalance that leads to maternal organ involvement, in contrast to the primarily fetoplacental restriction seen in isolated IUGR.

Similarly, the comparison between GAH and CAH revealed a profile centered on lipid metabolism, which is entirely distinct from these placental disorders. This confirms that despite their phenotypic similarity, GAH and CAH have different early-pregnancy etiologies. The GAH signature—marked by elevated *HRG*, *APCS*, *APOA4*, and *SERPINA6* and lower *APOC2* and *A1BG*—suggests a pregnancy-induced activation of metabolic and stress-response pathways, rather than a long-standing metabolic syndrome. This dynamic response is posited to occur in women with a latent vascular–metabolic vulnerability, who often present as young, primiparous, and without known risk factors [29]. The physiological stress of gestation unmasks this vulnerability, provoking the observed proteomic shifts [30]. In contrast, women with CAH exhibit a more stabilized profile, likely because pre-pregnancy treatment and ongoing medical surveillance have already attenuated part of their chronic metabolic burden.

The PLS-DA model visually confirmed this biological distinction, showing clear separation of PE from both GAH and CAH, while the latter two hypertensive conditions overlapped. However, the models developed here did not effectively discriminate between the CAH and GAH groups, presumably due to the shared clinical phenotype and the inherent diagnostic overlap between these conditions. The clinical distinction between chronic and gestational hypertension can be ambiguous, particularly in women with unrecognized preexisting hypertension, which may contribute to greater group homogeneity and reduced model separation.

The findings presented here consolidate and refine the existing proteomic landscape of PE, which has historically relied on semi-quantitative methods [14]. A substantial portion (40 out of 115) of the quantified proteins have been previously implicated as PE markers, as confirmed by recent meta-analysis [14], thus validating their involvement in the disease process. Notably, the direction of change for several key markers in this first-trimester cohort is aligns with studies from similar gestational windows. For instance, the observed decrease in AHSG and increase in AFM are consistent with findings from first- and second-trimester plasma studies [31,32]. Similarly, the downregulation of *FBLN1* [33], *SERPING1* [16], *KNG1* [17,33], alongside the upregulation of *RBP4* [33,34,35], *HPX* [36] and *TTR* [34], matches reports from third-trimester investigations of late-onset PE.

More importantly, critical temporal discrepancies were identified, revealing the dynamic nature of the maternal proteome. For several proteins, the alteration detected in the first trimester was the inverse of that reported at term. This is evident for *APCS* (elevated here versus decreased [33,37]), *F10* (decreased versus elevated [33]), *C1R* (decreased versus elevated [33]), and *APOA2* (decreased versus elevated [37]). These inversions highlight a fundamental principle: the first-trimester proteome likely reflects early, causative pathogenic processes, such as aberrant placentation, while the term proteome captures the downstream effects of systemic endothelial damage [28].

A detailed analysis suggests that inconsistencies in the literature often stem from differences in gestational age, PE subtype, and cohort size. For example, AFM was upregulated in this study and in a larger second-trimester cohort of mixed-onset PE [31], but downregulated in a smaller study of early-onset PE from the same research group [31]. Similar discrepancies exist for *FBLN1* and *VTN* [31,32,33]. These observations suggest that PE subtypes may have distinct, time-sensitive proteomic fingerprints. The conflicting reports for markers like *RBP4*, *HPX* and *TTR* across different third-trimester late-onset PE studies [16,34,35,36,37] further illustrate the complex interplay of these factors. Despite these complexities, the concordance of core PE markers—including *AFM*, *AHSG*, *FBLN1*, *KNG1*, *SERPING1*, *RBP4*, *TTR*, and *HPX*—with at least one independent study [14] reinforces their validity and central role in PE pathophysiology.

This study has several limitations. First, while the nested case–control design is powerful for discovery, it requires validation in a large, prospective, unselected cohort to confirm generalizability. Second, although women with major comorbidities were excluded, the presence of other common conditions, such as obesity or gestational diabetes diagnosed later in pregnancy, was not systematically excluded or adjusted for, which may influence the findings. Third, the single-center design and specific population may limit the transferability of the proteomic signature and its performance thresholds to other populations with different ethnic backgrounds, healthcare protocols, or baseline risk profiles. Fourth, the sample size, though substantial for a detailed proteomic study, limited the power of some subgroup analyses, particularly for IUGR. Finally, the targeted nature of the proteomic panel, though a strength for validation, confined the analysis to the 115-plex assay, precluding the discovery of novel biomarkers outside this panel. Future studies with discovery-phase proteomics could uncover additional biomarkers.

The immediate priority following this study is the validation of this proteomic signature in a large, prospective, unselected cohort. This is essential to confirm generalizability and define clinical risk thresholds. Concurrently, efforts should focus on translating the 18-protein signature into a simplified, cost-effective assay suitable for high-throughput clinical screening, paving the way for routine implementation.

Looking ahead, the current findings open two transformative paths for research. First, the distinct etiological pathways identified here warrant investigation into personalized prevention strategies, where at-risk women receive interventions tailored to their specific proteomic profile (e.g., complement- vs. lipid-dominant). Second, integrating these proteomic markers with established clinical predictors could create a supremely accurate combined model, while functional studies on key proteins like *AFM*, *AHSG*, *SERPING1*, *F10*, *APOA2* and *APOA4* are needed to advance from biomarker discovery to a mechanistic understanding of disease pathogenesis and novel therapeutic targets.

## 4. Materials and Methods

### 4.1. Study Design

This prospective cohort study was conducted at the V.I. Kulakov National Medical Research Center for Obstetrics, Gynecology and Perinatology between January and December 2022. The primary objective was to investigate the association between first-trimester serum protein biomarkers and the subsequent development of hypertensive disorders of pregnancy. The study initially enrolled 1869 Caucasian pregnant women aged 18 to 45 years who attended their routine first-trimester prenatal screening performed according to the FMF protocol at 11^+2^ to 14^+2^ weeks of gestation. Participants were excluded based on the following maternal or fetal conditions: multiple pregnancy; maternal diabetes, cancer, autoimmune disease, decompensated kidney disease, or organ transplant history; maternal congenital heart defects or arrhythmia; acute or exacerbated chronic infections; or confirmed fetal chromosomal abnormalities and congenital malformations. From the initial screening population, a final cohort of 1720 (Figure 1) women with singleton pregnancies was established. From this cohort, a nested case–control study was performed for analytical precision. Case groups were defined by the development of specific obstetric complications: PE (*n* = 32), GAH (*n* = 22), CAH (*n* = 24), and isolated IUGR (*n* = 11) without PE.

Hypertensive disorders were classified according to the 2021 International Society for the Study of Hypertension in Pregnancy (ISSHP) criteria [38]. CAH was defined as systolic blood pressure ≥ 140 mmHg and/or diastolic blood pressure ≥ 90 mmHg present before pregnancy or first detected before 20 weeks’ gestation. GAH was defined as new-onset systolic blood pressure ≥ 140 mmHg and/or diastolic blood pressure ≥ 90 mmHg arising after 20 weeks’ gestation in the absence of proteinuria or other maternal organ dysfunction. PE was defined as hypertension after 20 weeks’ gestation accompanied by one or more of the following: proteinuria; maternal organ dysfunction (renal, hepatic, hematologic, neurologic); or uteroplacental dysfunction, including fetal growth restriction.

A control group (*n* = 83) was selected from women who had uncomplicated singleton pregnancies, specifically excluding those who conceived via assisted reproductive technology or developed any hypertensive disorder, gestational diabetes (diagnosed by oral glucose tolerance testing), preterm delivery before 37 weeks, or IUGR (estimated fetal weight < 10th percentile).

Upon enrollment, written informed consent was obtained from all participants. A comprehensive first-trimester assessment was then conducted, which recorded standardized maternal weight, height, and blood pressure [39]. Ultrasonographic evaluation included transabdominal color Doppler measurement of the uterine artery pulsatility index (UtA-PI) [40]. Blood was drawn into Serum Z/9 tubes (Monovette, Sarstedt, Germany) and processed within 2 h of collection. After centrifugation during 20 min at room temperature with 300× *g*, the supernatant was aliquoted in vials. Serum was analyzed for PIGF and PAPP-A using the Delfia Xpress system (PerkinElmer, Shelton, CT, USA) as per the manufacturer’s guidelines. Residual serum from these assays was aliquoted in cryo-tubes and stored at −80 °C to facilitate a subsequent targeted proteomic analysis of 115 first-trimester biomarkers [20]. Pregnancies were followed up until delivery. Key clinical characteristics during pregnancy, pregnancy outcomes, and placental morphological features were systematically recorded.

The study protocol was approved by the Institutional Review Board of the National Medical Research Center for Obstetrics, Gynecology and Perinatology (protocol No. 2, dated 9 March 2017). All procedures were performed in accordance with the ethical standards of the Helsinki Declaration and Good Clinical Practice guidelines.

### 4.2. Sample Preparation

This study utilized a targeted proteomic approach with 115 stable isotope-labeled (SIS) peptides as internal standards and their corresponding 115 unlabeled (NAT) analogues for calibration. All SIS and NAT peptides were synthesized and characterized in the Omics lab at Skoltech according to established protocols [41,42]. The assay was adapted from the commercially available BAK-270 kit (MRM Proteomics, Montreal, QC, Canada) [20,43].

Sample preparation followed standard procedures [20,41,44]. Briefly, 10 µL of serum was used for each sample. Proteins were denatured and reduced in a solution containing 9 M urea, 20 mM dithiothreitol, and 200 mM Tris × HCl (pH 8.0) for 30 min at 37 °C, followed by alkylation with 100 mM iodoacetamide for 30 min in the dark. For tryptic digestion, samples were diluted with 100 mM Tris × HCl (pH 8.0) to reduce the urea concentration below 1 M, incubated with TPCK-trypsin at a 20:1 protein-to-enzyme ratio, and digested for 18 h at 37 °C. The digestion was quenched by adding formic acid (FA) to a final concentration of 1.0% (pH ≤ 2), yielding a peptide concentration of approximately 1 mg/mL.

A uniform aliquot of the SIS peptide mixture was added to all samples—including the 172 clinical samples, calibration standards, and quality controls—at a final concentration of 100 fmol/µL in 10 µL. In contrast, the NAT peptides were added only to the seven-point calibration curve (levels A–G) at concentrations spanning from the lower limit of quantification (LLOQ, level A) to the upper limit of quantification (HLOQ, level G). Following the addition of SIS peptides, all samples underwent solid-phase extraction (SPE) cleanup. The resulting peptides were reconstituted in 34 µL of 0.1% FA for subsequent LC-MRM-MS analysis.

Robust quality control was implemented using two distinct types of QC samples. The BSA-based QCs (QCA-C), prepared in a surrogate matrix at low, medium, and high concentrations, were used to monitor assay performance. A pooled serum QC (CLP) was included to track batch-to-batch reproducibility. Process blanks were analyzed to assess potential background contamination. All calibration standards and QC samples were processed identically to the clinical serum samples.

### 4.3. LC-MRM-MS Analysis

LC-MRM-MS analyses were performed in three randomized batches on an ExionLC UHPLC system (ThermoFisher Scientific, Waltham, MA, USA), interfaced with a SCIEX QTRAP 6500+ mass spectrometer (SCIEX, Toronto, ON, Canada). All 177 clinical samples were measured in randomized duplicate. The analytical sequence for each batch was designed to include a complete set of seven-point calibration standards, quality controls (BSA-based QCA-C and pooled serum CLP), and process blanks on every 96-well plate.

Peptide separation was carried out on a C18 column (2.1 × 150 mm, 1.7 μm) using a 53-min linear gradient of 2–45% acetonitrile in 0.1% FA at a constant flow rate of 0.4 mL/min [20,42,43]. The mass spectrometer was operated in positive electrospray ionization mode with a source voltage of 4000 V and temperature of 450 °C. Data acquisition utilized multiply reaction monitoring, with the specific precursor/product ion transitions provided in Table S18.

Robust quality assurance was embedded in the batch design. Each batch commenced with the calibration curve, while QC samples were interspersed at the beginning, middle, and end of the run to assess system stability and quantitative reproducibility throughout the entire analysis.

### 4.4. Data Preprocessing

Raw mass spectrometry data were processed and quantified using Skyline software (version 20.2.0.343) [45]. Quantification and data quality assessment adhered to the principles outlined in the ICH guidelines for Bioanalytical Method Validation [46]. Calibration curves were constructed from the seven-point standard series (levels A–G) using a 1/x^2^-weighted linear regression model to calculate peptide and corresponding protein concentrations in fmol/µL of plasma.

Assay performance was monitored using calibration standards and QC samples (QCA-C and CPL). The acceptance criteria mandated that the accuracy and precision of calculated concentrations be within ±20% of their theoretical values. A calibration curve was accepted if at least five of its seven points met these criteria. For an entire analytical batch to be considered valid, a minimum of 66% of all QC samples and no less than 90% of all peptide calibration curves were required to pass the acceptance criteria. Proteins with a calibration curve demonstrating an R^2^ value greater than 0.99 were classified as “quantified” (Figure 1).

For study samples, measured concentrations below the lower limit of quantification (LLOQ, level A) were imputed with the LLOQ value, while those above the upper limit of quantification (HLOQ, level G) were assigned the HLOQ value. Furthermore, any protein with concentrations outside the quantifiable range (below LLOQ or above HLOQ) in more than 50% of the study samples was excluded from subsequent analysis.

### 4.5. Statistical Analysis

Data normalization was performed using the RobNorm method [47] to mitigate batch effects and analytical variance, consistent with a previous study of first-trimester serum proteomics in healthy pregnancy [20]. For each protein, the mean CV was calculated across standard and quality control samples between batches. Proteins exhibiting a CV greater than 50% were excluded from subsequent analysis. Furthermore, protein expression values were adjusted using MoM derived from the aforementioned reference study and subsequently adjusted for maternal age, body mass index, parity, fetal sex, the presence of uterine myomas, and gestational age at blood draw, based on models established in the same prior work [20].

The analysis focused on comparing specific pairs of pregnancy complications: PE versus isolated IUGR; GAH versus CAH; GAH versus PE; and PE versus all non-PE cases, which included CAH, GAH, the control group, and the IUGR group. Clinical parameters between each pair were compared using the Mann–Whitney U test for continuous variables and Pearson’s chi-square test for categorical variables, with an alteration deemed statistically significant if the *p*-value was less than 0.05 and the test power exceeded 0.80. For an omnibus comparison across all five groups (Control, CAH, GAH, IUGR, PE), the Kruskal–Wallis test and Pearson’s chi-square test were employed, followed by Dunn’s test and pairwise chi-square tests with Bonferroni correction for post hoc analysis when the initial *p*-value was below 0.05.

For each comparative pair, discriminant analysis was conducted using OPLS-DA [48]. Models were considered to have good discrimination and predictive quality with R^2^Y > 0.5 and Q^2^Y > 0.4, respectively. Protein levels were also compared between groups using the Mann–Whitney U test. Potential protein markers for a complication were identified by combining a *p*-value < 0.05 from this test with a variable importance in projection (VIP) score greater than 1 from the OPLS-DA [49] (Figure 1). A new OPLS-DA model was then built using only these marker proteins. For each resulting marker set, protein–protein interaction networks and metabolic pathway enrichment analyses were performed using the STRING resource [50]. Pathways with a false discovery rate (FDR) below 0.01 were considered significantly enriched, and a final enrichment score was calculated as the weighted harmonic mean of the observed-to-expected protein ratio and the −log(FDR).

The proteomic associations between CAH, GAH, and PE were further explored using Partial Least Squares-Discriminant Analysis (PLS-DA) [51], with proteins classified as markers for each condition based on their loadings on latent components, prioritizing those with a VIP > 1. Associations between clinical parameters and the previously identified protein markers were assessed using Spearman’s rank correlation with a significance threshold of *p* = 0.05.

Finally, the diagnostic potential of the identified protein markers was evaluated by constructing several classification models, including OPLS-DA, SVM with linear, polynomial, radial, and sigmoid kernels, XGBoost, and a MLP. Hyperparameters for the non-linear SVM kernels, XGBoost, and MLP were optimized using a particle swarm optimization method with 5-times repeated 5-fold cross-validation (Figure 1). Model performance was evaluated through 10-fold cross-validation to determine final sensitivity, specificity, and accuracy. The resulting classifier was then compared directly to the routine FMF-based test. For this comparison, PPV and NPV were calculated using the established global prevalence of PE (4%) [52].

All statistical analyses were performed using R version 4.3.2 [53] within RStudio 2023.09.1 [54]. The analysis leveraged numerous specialized packages including ropls 1.34.0 [55], effsize 0.8.1 [56], dunn.test 1.3.6 [57], jgsbook 1.0.7 [58], Desctools 0.99.60 [59], pwr 1.3-0 [60], xgboost 1.7.8.1 [61], e1071 1.7-16 [62], caret 7.0-1 [63], dplyr 1.1.4 [64] and keras 2.15.0 [65] for statistical modeling and machine learning, while ggplot2 3.5.2 [66], reshape2 1.4.4 [67], forcats 1.0.0 [68], ggrepel 0.9.6 [69] and pROC 1.18.5 [70] were used for data visualization.

## 5. Conclusions

In conclusion, this study provides robust evidence that first-trimester maternal serum proteomics can serve as a powerful tool for the early prediction and discrimination between hypertensive pregnancy disorders. The identified protein signatures point to specific underlying biological pathways—primarily dysregulated complement and coagulation systems in PE and lipid metabolism in GAH—that are active long before clinical manifestation. The developed 18-protein model demonstrates a level of diagnostic accuracy that surpasses current screening methods, holding promise for a paradigm shift towards a more precise, etiology-based risk assessment in the first trimester. This would enable targeted monitoring and potentially early, preventative interventions for women at highest risk for preeclampsia, while reassuring those at low risk.

## Figures and Tables

**Table 1:** Pearson correlation analysis of first-trimester serum PAPP-A levels quantified by ELISA and LC-MRM-MS.

ELISA (FMF Screening)	LC-MRM-MS	R	*p*-Value
0.96 (0.65; 1.48) MoM	5.54 (3.80; 8.03) mM	0.61	*p* < 0.001
0.96 (0.65; 1.48) MoM	0.80 (0.47; 1.16) MoM	0.60	*p* < 0.001

**Table 2:** Quality of OPLS-DA discrimination models, based on all proteins and sets of proteins features.

	All Proteins			Proteins-Markers		
	R^2^X	R^2^Y	Q^2^Y	R^2^X	R^2^Y	Q^2^Y
IUGR vs. PE	0.46	0.99	0.46	0.57	0.76	0.67
CAH vs. GAH	0.19	0.89	0.14	1	0.64	0.55
GAH vs. PE	0.25	0.97	0.72	0.47	0.82	0.77
Non-PE vs. PE	0.14	0.89	0.79	0.62	0.84	0.8

**Table 3:** The lists serum proteins that demonstrated significant alterations (VIP > 1 and *p* < 0.05) in first trimester identified by PLS-DA models. Arrows denote the direction of protein level’s change in at least one comparative group: ↓ decrease; ↑ increase.

Protein Name	Gene Name	PE vs. IUGR	GAH vs. CAH	PE vs. GAH	PE vs. Non-PE
Afamin	*AFM*	-	-	-	↑
Alpha-1B-glycoprotein_VAR_018369	*A1BG*	-	↓	-	-
Alpha-2-HS-glycoprotein	*AHSG*	↓	-	↓	↓
Apolipoprotein A-II	*APOA2*	-	-	-	↑
Apolipoprotein A-IV	*APOA4*	-	↑	-	-
Apolipoprotein C-II	*APOC2*	-	↓	-	-
Beta-Ala-His dipeptidase	*CNDP1*	-	-	-	↑
Coagulation factor X	*F10*	↓	-	↓	↓
Complement C1q subcomponent subunit A	*C1QA*	↓	-	↓	↓
Complement C1q subcomponent subunit C	*C1QC*	↓	-	↓	↓
Complement C1r subcomponent	*C1R*	-	-	-	↓
Complement C1r subcomponent-like protein	*C1RL*	-	-	↑	-
Complement component C8 alpha chain	*C8A*	↓	-	↓	↓
Corticosteroid-binding globulin	*SERPINA6*	-	↑	-	-
Fibulin-1	*FBLN1*	-	-	-	↓
Ficolin-3	*FCN3*	↑	-	-	-
Hemopexin	*HPX*	-	-	↑	↑
Histidine-rich glycoprotein	*HRG*	-	↑	-	-
Ig gamma-1 chain C region	*IGHG1*	↑	-	-	↑
Kininogen-1	*KNG1*	-	-	↓	↓
Plasma protease C1 inhibitor	*SERPING1*	↑	-	↑	↑
Retinol-binding protein 4	*RBP4*	-	-	-	↑
Serum amyloid P-component	*APCS*	-	↑	-	↑
Transthyretin	*TTR*	↑	-	↑	↑
Vascular cell adhesion protein 1	*VCAM1*	-	-	↓	-
Vitronectin	*VTN*	↑	-	↑	↑

**Table 4:** Diagnostic performance of optimal classification models for pregnancy disorders. Metrics include sensitivity, specificity, accuracy, positive predictive value (PPV), negative predictive value (NPV), and F1-score.

Task	Proteins	Model	Sens.	Spec.	Acc.	PPV	NPV	F_1_-Score
IUGR vs. PE	F10, C8A, C1QA, AHSG, C1QC, SERPING1, TTR, IGHG1, VTN, FCN3	SVM, pol. kernel (degree = 3.7, γ = 1.9 × 10^−7^, coef_0_ = −310)	0.94	1	0.95	1	0.85	0.97
		SVM, rad. kernel (γ = 0.02)						
CAH vs. GAH	HRG, APCS, APOA4, SERPINA6, APOC2 A1BG	OPLS-DA	0.91	0.92	0.91	0.91	0.92	0.91
GAH vs. PE	TTR, SERPING1, HPX, VTN, C1RL, F10, C8A, AHSG, C1QA, C1QC, VCAM1, KNG1	SVM, rad. kernel (γ = 0.01)	0.95	0.94	0.94	0.97	0.91	0.96
PE vs. non-PE	F10, C8A, AHSG, C1QA, C1QC, FBLN1, KNG1, C1R, VTN, AFM, HPX, APOA2, RBP4, APCS, CNDP1, TTR, IGHG1, SERPING1	SVM, pol. kernel (degree = 3.6, γ = 1.3 × 10^−6^, coef_0_ = −70.4)	0.94	1	0.99	1	0.99	0.97
		SVM, rad. kernel (γ = 3.8 × 10^−3^)						

**Table 5:** Prediction of PE by the protein-based model versus the standard FMF first-trimester screening.

Clinical Group	No PE		PE	
	Proteome-Based Model	FMF	Proteome-Based Model	FMF
Control (*n* = 83)	83	82	0	1
CAH (*n* = 24)	24	17	0	7
GAH (*n* = 22)	22	17	0	5
IUGR (*n* = 11)	11	11	0	0
PE (*n* = 32)	2	15	30	17

## Data Availability

The original contributions presented in this study are included in the article/Supplementary Materials. Further inquiries can be directed to the corresponding author.

## Supplementary Materials

The following supporting information can be downloaded at: https://www.mdpi.com/article/10.3390/ijms27031402/s1.

## Abbreviations

The following abbreviations are used in this manuscript:

BMI	Body mass index
CAH	Chronical arterial hypertension
CS	Cesarean section
CV	Coefficient of variation
DAP	Diastolic arterial pressure
GAH	Gestational arterial hypertension
HLOQ	The highest limit of quantification
IUGR	Intrauterine growth restriction
LC-MS	Liquid chromatography-mass spectrometry
MRM	Multiple reaction monitoring
LLOQ	The lowest limit of quantification
MAP	Mean arterial pressure
MoM	Multiply of medians
MS	Mass spectrometry
NAT	Natural synthetic proteotypic peptides
OPLS-DA	Orthogonal projections on latent structure discriminant analysis
PE	Preeclampsia
PSO	Particle swarm optimization
SIS	Stable isotope-labeled standards
SVM	Support vector machine
VIP	Variable importance projection
QC	Quality control
