# Supplementary material for: Machine Learning and Blood-Targeted Proteomics Enable Early Prediction and Etiological Discrimination of Hypertensive Pregnancy Disorders
Source: Int J Mol Sci. 2026 Jan 30;27(3):1402. doi: 10.3390/ijms27031402 (PMC12897837; doi:10.3390/ijms27031402)
Supplement: Supplementary file 1 [file ijms-27-01402-s001.zip › ijms-4103917-supplementary/Supplementary figures.pdf]

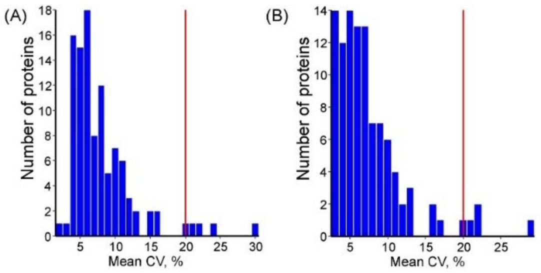

Figure S1. Assessment of batch effect correction. Distribution of the mean between-batch coefficient of variation (CV) across the proteome for (A) raw data and (B) data after RobNorm normalization.

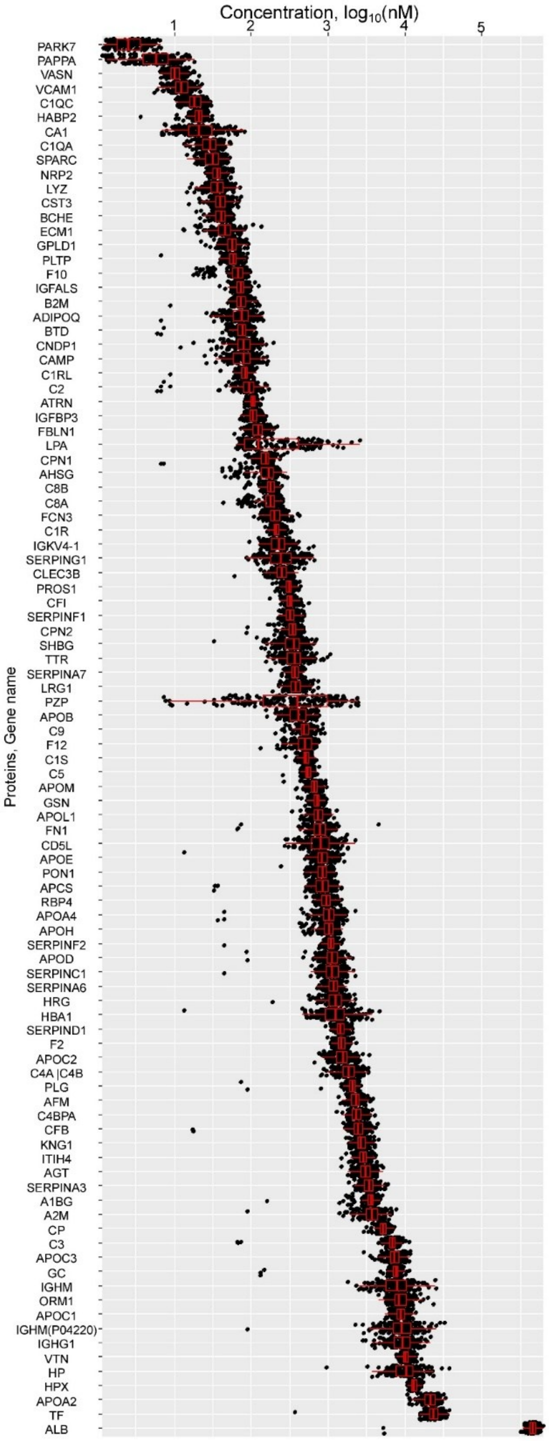

Figure S2. Distribution of protein concentrations after RobNorm normalization.

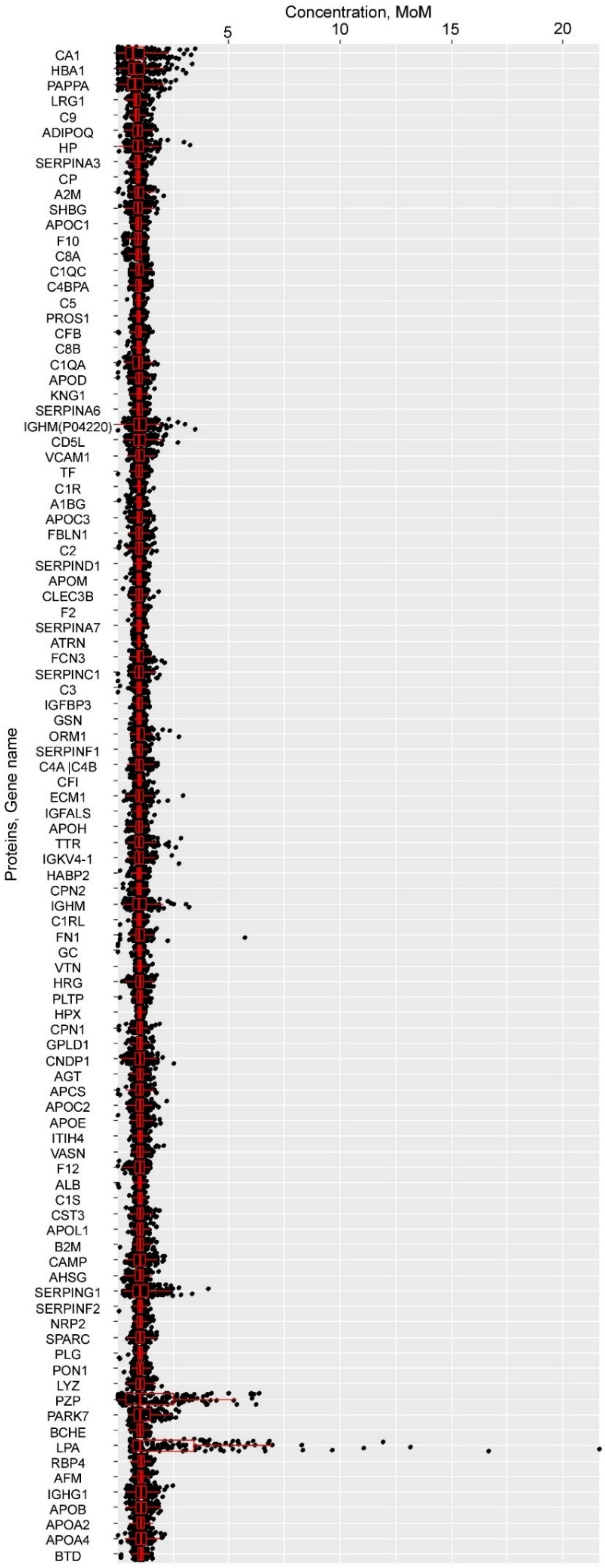

Figure S3. Distribution of protein levels after clinical adjustment. Protein concentrations were normalized to Multiples of the Median (MoM) to correct for clinical covariates.

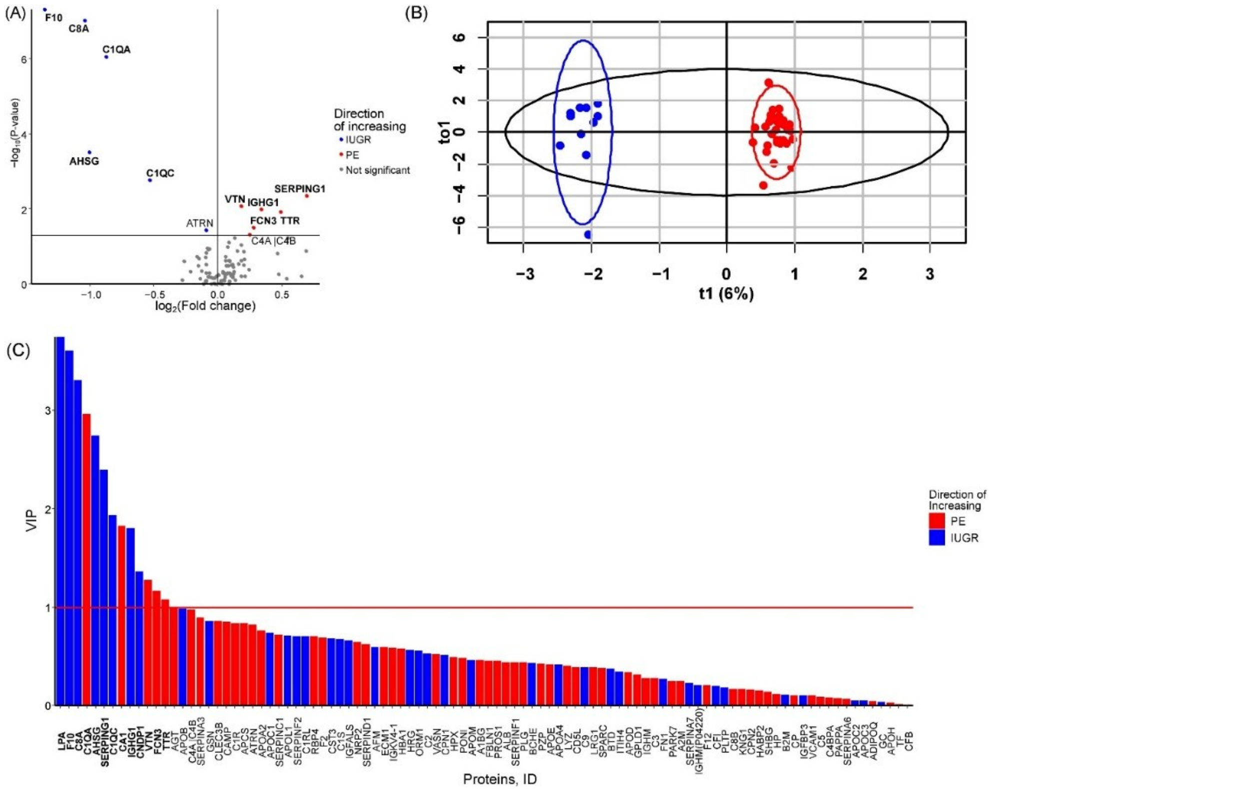

Figure S4. Proteomic analysis of PE vs. IUGR. (A) Volcano plot displaying protein alterations between PE and IUGR groups. The fold change (FC) is calculated as the ratio of median protein concentration (PE/IUGR). P-values were determined by the Mann-Whitney test. Final protein markers are highlighted in bold. (B) OPLS-DA score plot discriminating the IUGR (blue) and PE (red) groups. (C) Variable Importance in Projection (VIP) scores for proteins in the OPLS-DA model. Proteins with VIP > 1, considered major discriminators, are labeled in bold. The red line indicates the VIP=1 threshold.

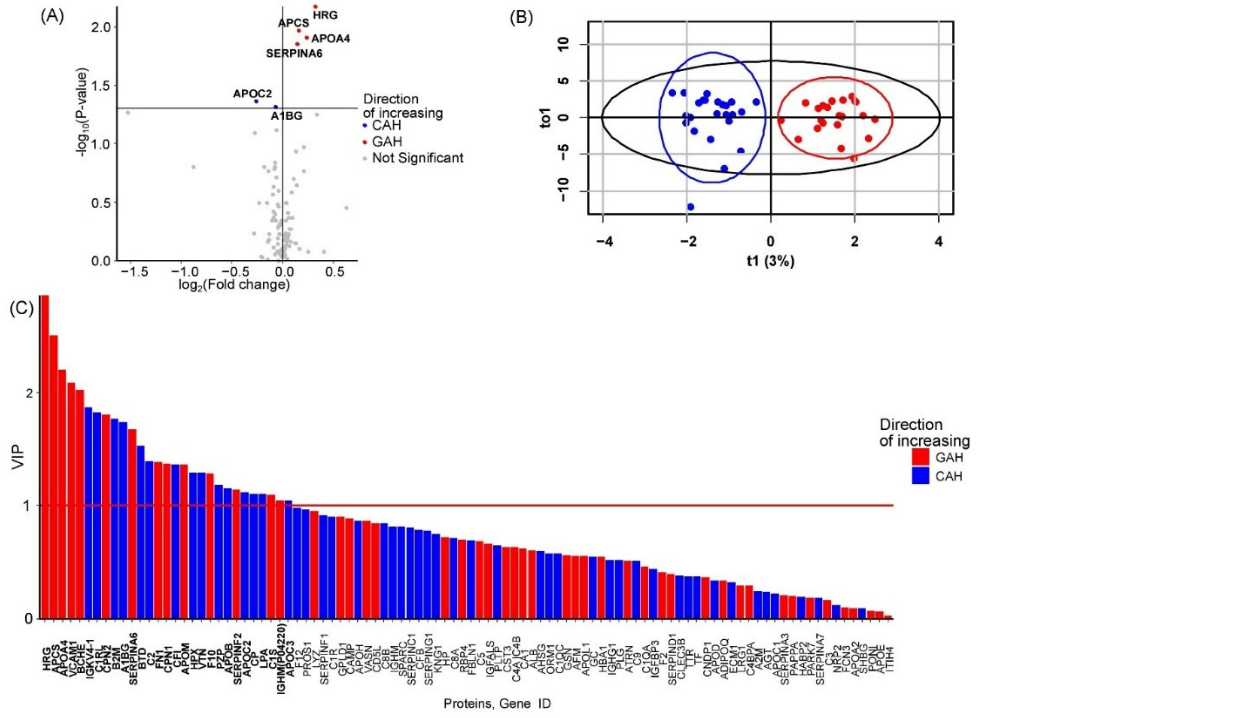

Figure S5. Proteomic analysis of GAH vs. CAH. (A) Volcano plot of protein alterations between GAH and CAH groups (FC: GAH/CAH; Mann-Whitney test). Final markers are bolded. (B) OPLS-DA score plot discriminating CAH (blue) and GAH (red) groups. (C) VIP scores from the OPLS-DA model. Proteins with VIP > 1 are bolded, with the red line marking the threshold.

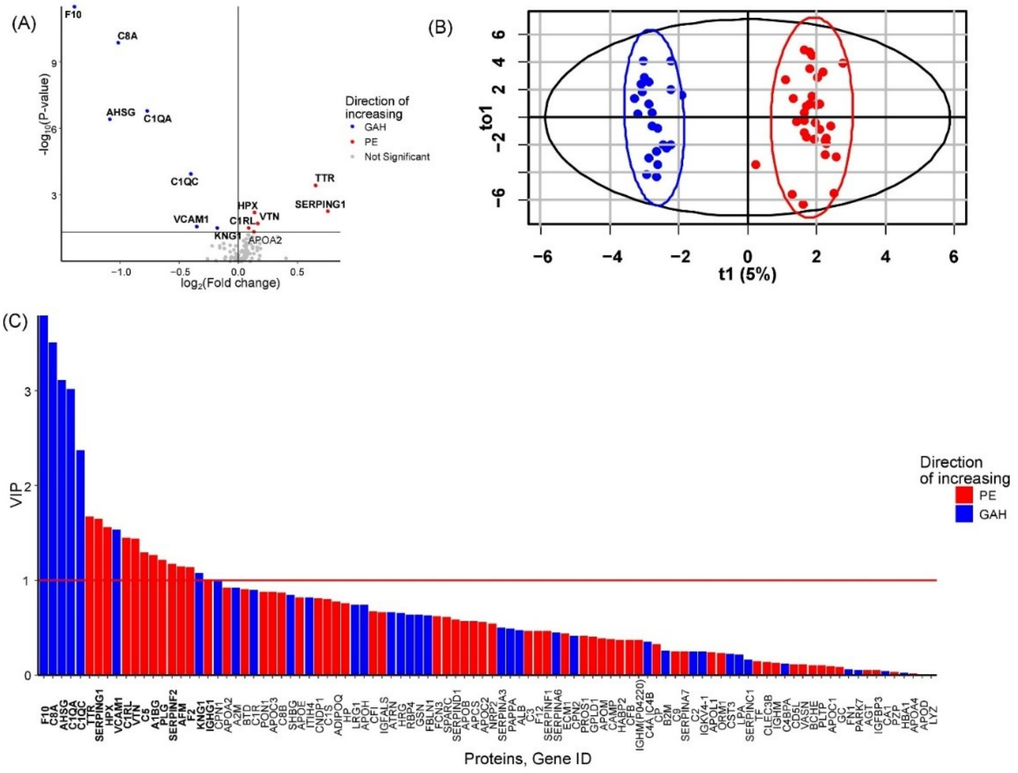

Figure S6. Proteomic analysis of PE vs. GAH. (A) Volcano plot of protein alterations between PE and GAH groups (FC: PE/GAH; Mann-Whitney test). Final markers are bolded. (B) OPLS-DA score plot discriminating GAH (blue) and PE (red) groups. (C) VIP scores from the OPLS-DA model. Proteins with VIP > 1 are bolded, with the red line marking the threshold.

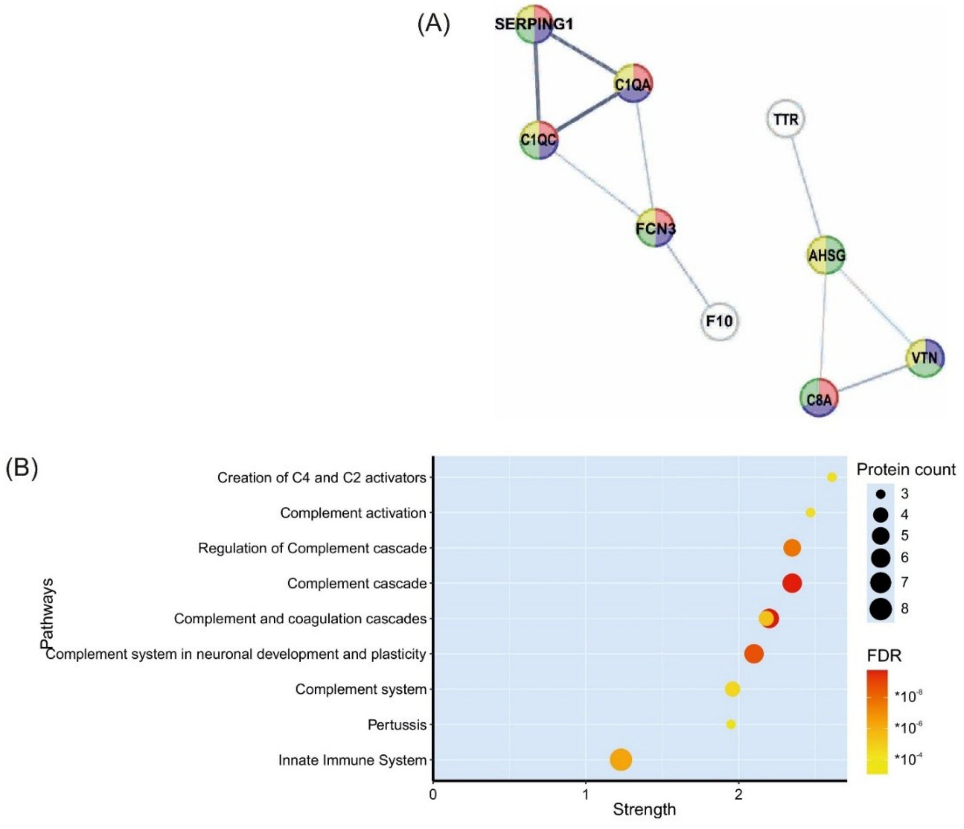

Figure S7. Functional analysis of protein markers discriminating PE from IUGR. (A) Protein-protein interaction (PPI) network from STRING database. Proteins are color-coded by biological process or cellular component: complement activation (red), immune response (blue), blood microparticles (green),

and collagen-containing extracellular matrix (yellow). (B) The top 10 most significantly enriched pathways. Point size corresponds to the number of proteins in the pathway, and color represents the false discovery rate (FDR).

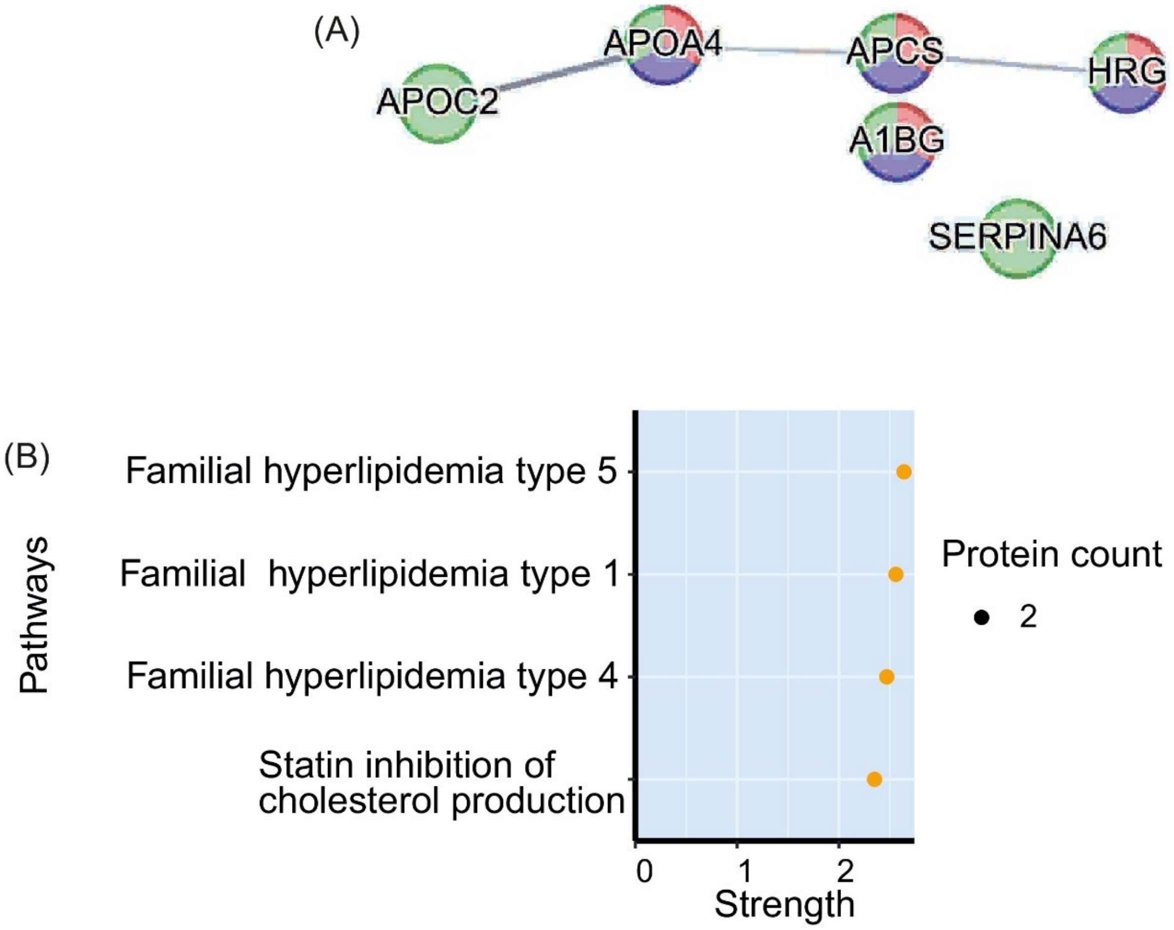

Figure S8. Functional analysis of protein markers discriminating GAH from CAH. (A) PPI network from STRING. Proteins are color-coded by cellular component: blood microparticles (red), collagen-containing extracellular matrix (blue), and extracellular space (green). (B) Statistically significant enriched pathways. Point size corresponds to the number of proteins in the pathway.

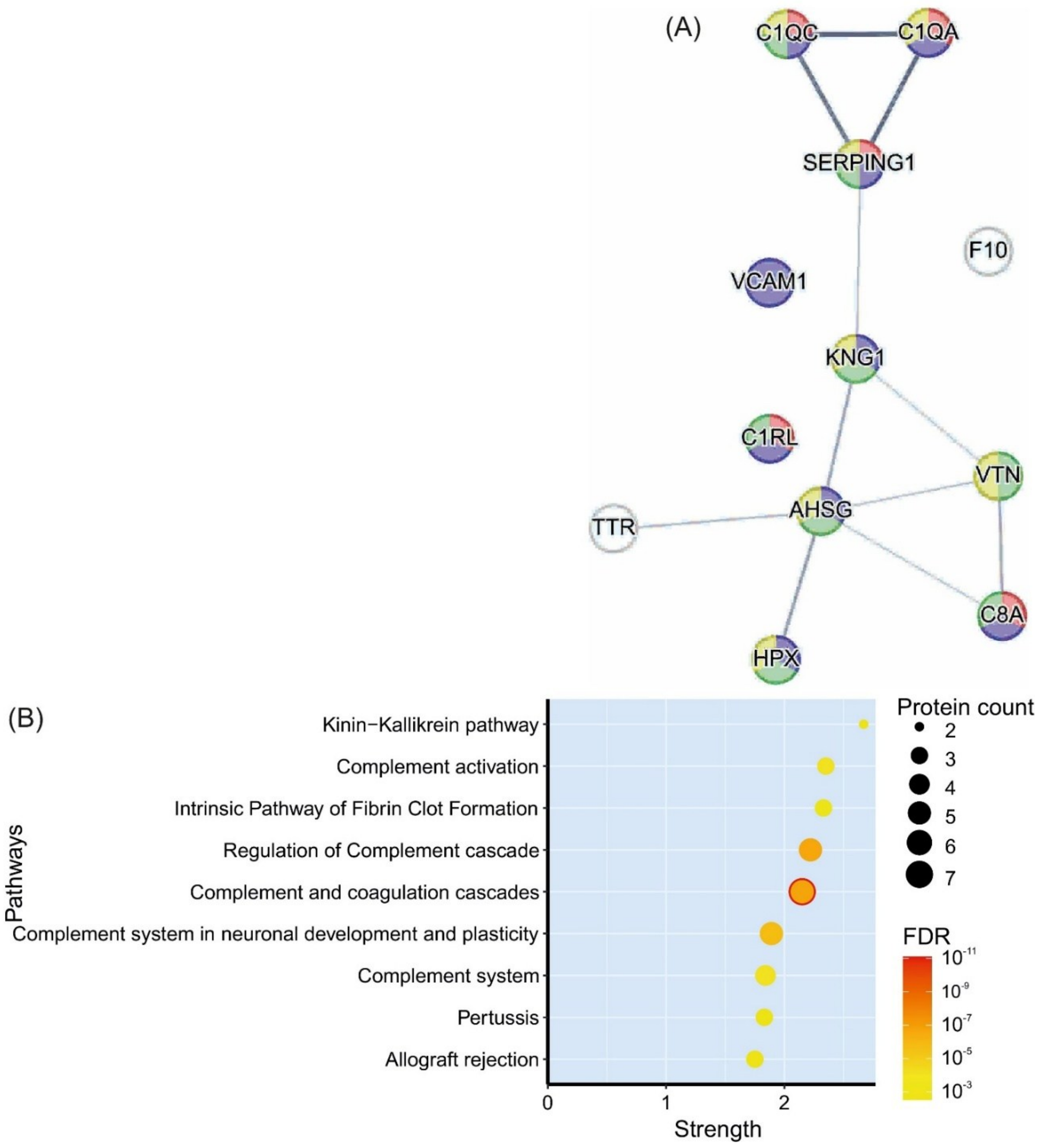

Figure S9. Functional analysis of protein markers discriminating PE from GAH. (A) PPI network from STRING. Proteins are color-coded by biological process or cellular component: complement activation (red), defense response (blue), blood microparticles (green), and collagen-containing extracellular matrix (yellow). (B) The top 10 most significantly enriched pathways. Point size corresponds to the number of proteins in the pathway.

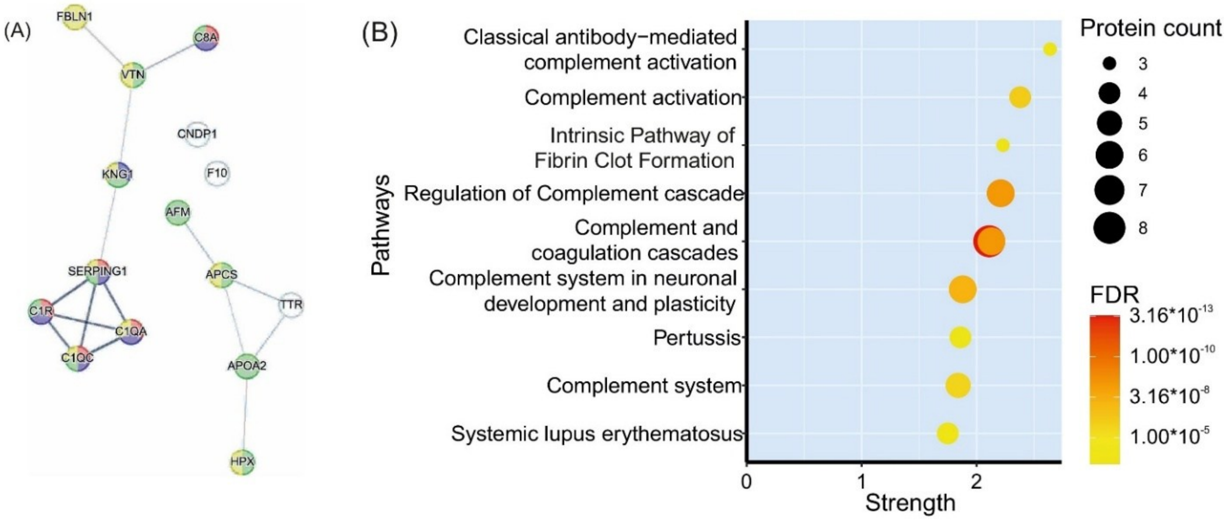

Figure S10. Functional network and pathway enrichment of first-trimester serum PE-specific protein panel. An interaction network generated using STRING, displaying proteins that discriminate PE cases (n=32) from non-PE (n=142). Node colors indicate functional and locational groups: complement activation (red), humoral immune response (blue), blood microparticles (green), and collagen-containing extracellular matrix (yellow). B. Bubble plot of the top ten most significantly enriched pathways. The size of each bubble is proportional to the number of marker proteins mapped to the respective pathway.

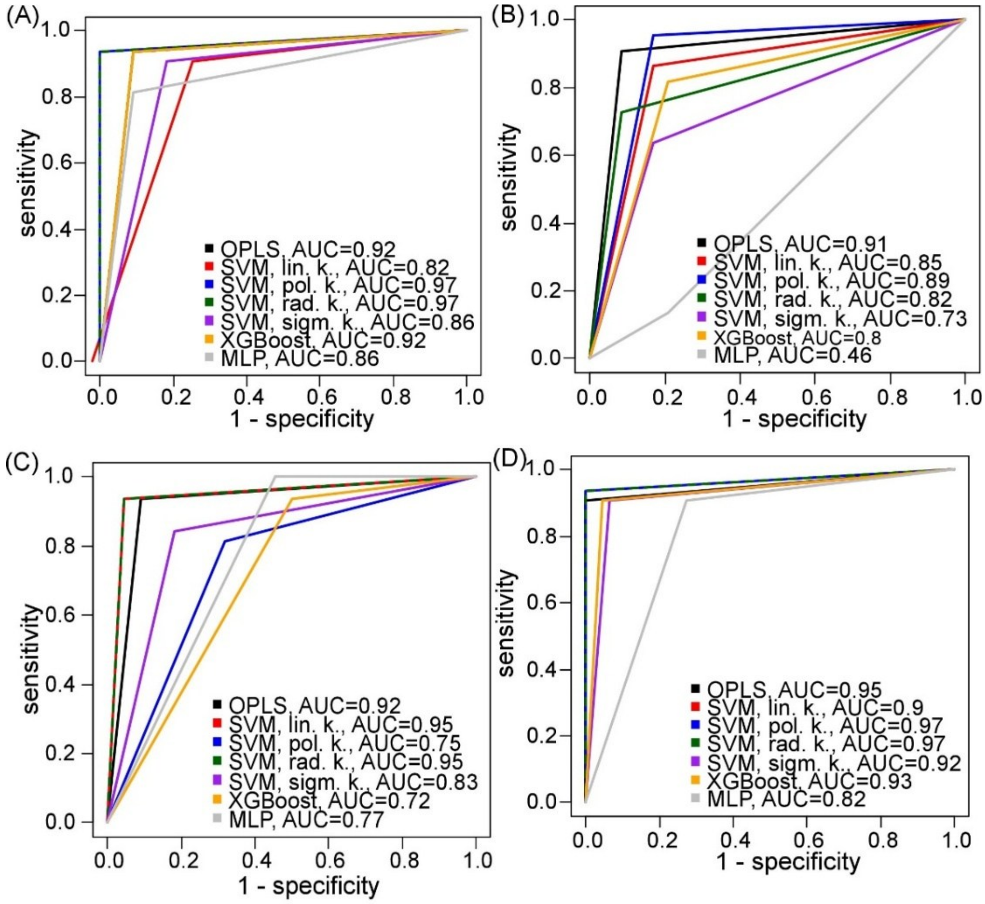

Figure S11. Diagnostic performance of classification models for pregnancy disorders. ROC curves generated during cross-validation for discriminating: (A) IUGR (n=11) and PE (n=32) cases; (B) CAH (n=24) and GAH (n=22) cases; (C) GAH (n=22) and PE (n=32) cases; (D) PE (n=32) cases from all non-PE outcomes (n=140).

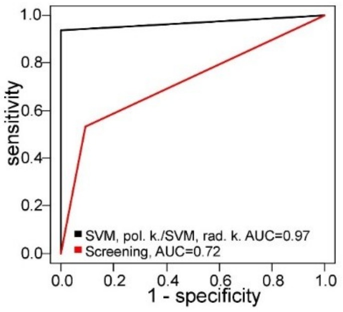

Figure S12. Comparison of first-trimester prediction models for PE. Receiver operating characteristic (ROC) curves of the support vector machine (SVM) proteomic model versus the standard FMF screening model for discriminating PE from all other non-PE groups (Control, CAH, GAH, and IUGR).

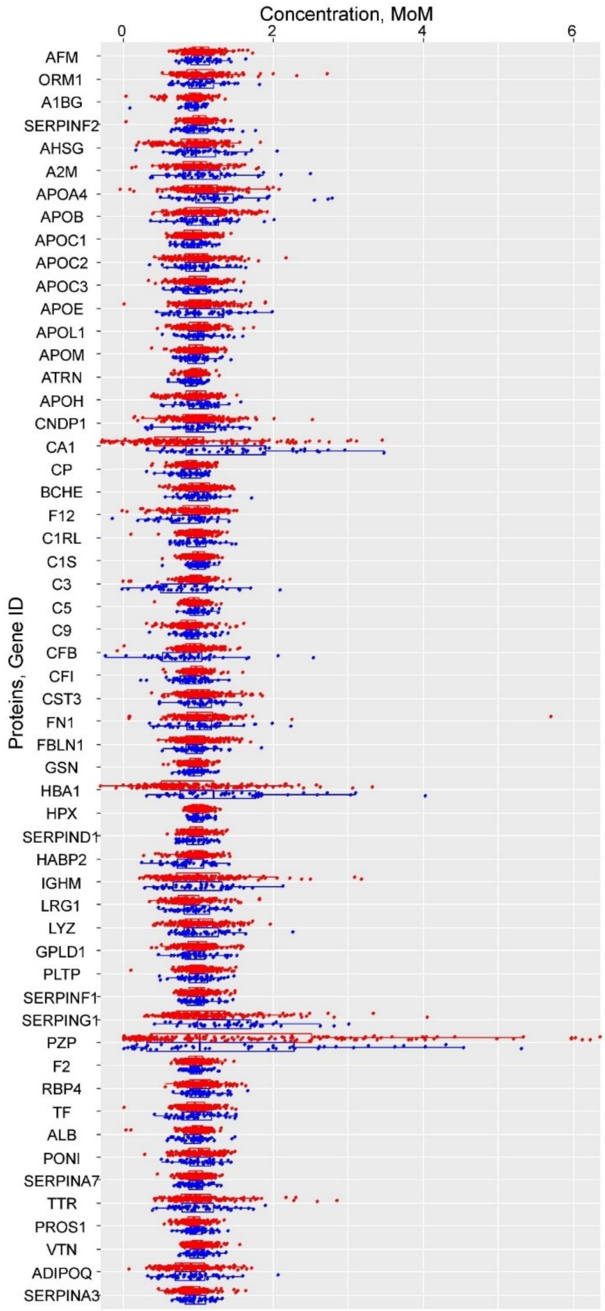

Figure S13. Cross-validation of first-trimester serum protein levels. Distribution of normalized and clinically-adjusted protein concentrations from the current study (red) compared to the pilot study (blue; reference [21]).

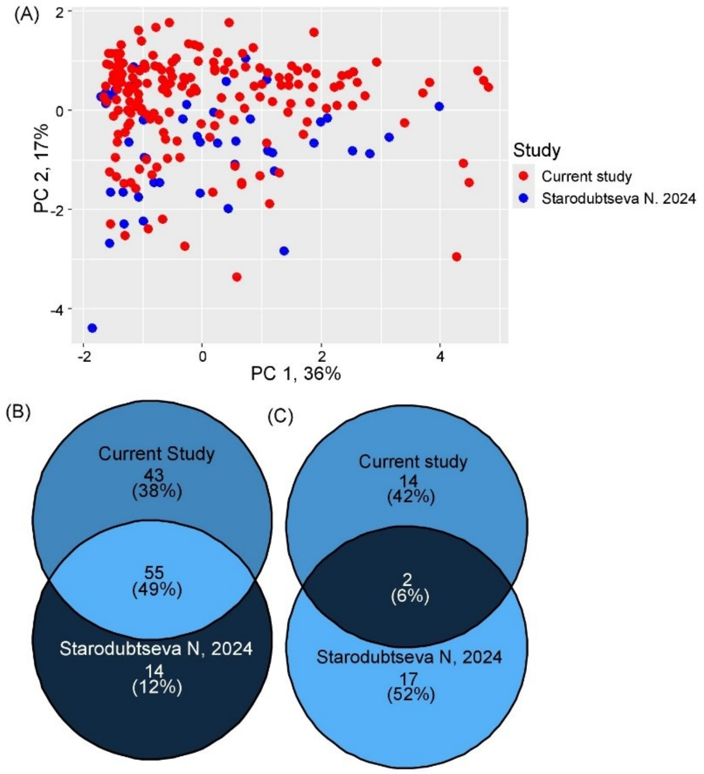

Figure S14. Analytical and model concordance in external validation. (A) PCA showing overlap between the current cohort (n=172, red) and the validation cohort [21] (n=40, blue). (B) Overlap of quantified proteomic features. (C) Shared and unique proteins incorporated into the PE diagnostic models of each study.
